# Viewing an alpine environment positively affects emotional analytics in patients with somatoform, depressive and anxiety disorders as well as in healthy controls

**DOI:** 10.1186/s12888-020-02787-7

**Published:** 2020-07-23

**Authors:** Katharina Hüfner, Cornelia Ower, Georg Kemmler, Theresa Vill, Caroline Martini, Andrea Schmitt, Barbara Sperner-Unterweger

**Affiliations:** 1 Department of Psychiatry, Psychotherapy and Psychosomatics, Divison of Psychiatry II (Psychosomatic Medicine), Medical University Innsbruck, Anichstr. 35, 6020 Innsbruck, Austria; 2 Department of Psychiatry, Psychotherapy and Psychosomatics, Divison of Psychiatry I, Medical University Innsbruck, Innsbruck, Austria; 3 Department of Psychiatry and Psychotherapy, University Hospital, Ludwig Maximilians-University (LMU) Munich, Munich, Germany; 4 Laboratory of Neuroscience (LIM27), Institute of Psychiatry, University of São Paulo, São Paulo, Brazil

**Keywords:** Alpine environment, Resilience, Self-perceived stress, Self-Assessment Manikin, Emotional analytics, Somatoform, depressive and anxiety disorders

## Abstract

**Background:**

Patients with somatoform, depressive or anxiety disorders often don’t respond well to medical treatment and experience many side effects. It is thus of clinical relevance to identify alternative, scientifically based, treatments. Our approach is based on the recent evidence that urbanicity has been shown to be associated with an increased risk for mental disorders. Conversely, green and blue environments show a dose-dependent beneficial impact on mental health.

**Methods:**

Here we evaluate the effect of viewing stimuli of individuals in an alpine environment on emotional analytics in 183 patients with psychiatric disorders (mostly somatoform, depressive and anxiety disorders) and 315 healthy controls (HC). Emotional analytics (valence: unhappy vs happy, arousal: calm vs excited, dominance: controlled vs in control) were assessed using the Self-Assessment Manikin. Further parameters related to mental health and physical activity were recorded.

**Results:**

Emotional analytics of patients indicated that they felt less happy, less in control and had higher levels of arousal than HC when viewing neutral stimuli. The comparison alpine>neutral stimuli showed a significant positive effect of alpine stimuli on emotional analytics in both groups. Patients and HC both felt attracted to the scenes displayed in the alpine stimuli. Emotional analytics correlated positively with resilience and inversely with perceived stress.

**Conclusions:**

Preventive and therapeutic programs for patients with somatoform, depressive and anxiety disorders should consider taking the benefits of natural outdoor environments, such as alpine environments, into account. Organizational barriers which are preventing the implementation of such programs in clinical practice need to be identified and addressed.

## Background

The natural environment is known to improve physical and mental health: A meta-analysis reported an 8% reduction in all-cause mortality for residents with the highest nature outdoor exposure compared with the lowest exposure group [14]. Discovering blue [11] and green [43] spaces is associated with psychological benefits. Stress partly mediates the effect of natural outdoor environments on mental well-being [39]. Green spaces have been shown to reduce cortisol levels as a marker of stress [40]. Stress as an important marker of mental health is significantly reduced by the exposure to nature in a dose-response relationship, even if only visual stimulation without physical exposure is used [17]. Visual or auditory nature stimuli can facilitate recovery from psychological stressful events [1, 5] and from physical disease [41]. In mental health, chronic stress is among the strongest risk factors for depression but is also an important pathogenetic factor in anxiety disorders, post-traumatic stress disorders or somatoform disorders [2, 35].

Another factor through which exposure to natural outdoor environments exerts its positive effect on mental health might be through the strengthening of resilience [33, 34]. Resilience can be defined as one’s ability to cope with and recover from adverse life events. Resilience is improved by physical activity performed in a natural outdoor environment but is not associated with physical activity performed indoors [32]. When the natural environment is used to perform physical activity the positive effects of physical activity and natural environments can be combined: there is evidence that exercising outdoors results in greater improvements of mental well-being than exercising indoors with greater feelings of delight, energy and revitalization, as well as decreases in frustration, tiredness and anger [38].

The positive effects of the alpine natural environment have rarely been examined. One of the few available studies suggests that watching grand mountain scenes triggers a greater mood improvement than mundane nature. Furthermore, participants were feeling significantly more connected to others, more caring, and more spiritual after watching awe-inspiring nature condition [20]. Hikers of alpine wilderness trails reported substantial stress reduction and mental rejuvenation following a day or overnight hike [8]. Furthermore, in a crossover trial focusing on differences between indoor and alpine activity, mountain hiking showed significantly greater positive effects on affective valence and activation compared to indoor physical activity [29]. It is unknown whether the mechanisms linking different natural environments (green space, blues space, alpine) to mental health are due to similar or differential effects [13, 28].

Although studies report an improvement on various psychological measures as a result of exposure to alpine environments, they do not refer to a possible therapeutic effect. There are only few studies investigating therapeutic alpine interventions as treatment for patients in mental health care. In a mountain hiking program for suicidal patients, participants reported significant reduction in depression, hopelessness and suicidal ideation [37]. In another study adults and youth with mental illness experienced significant improvements in self-esteem, mastery and resilience following activities like mountain biking and raft building [4].

The primary aim of the present study was to investigate whether stimuli depicting alpine environments would elicit differential or similar emotional analytics in patients with somatoform, depressive and anxiety disorders and healthy controls in order to judge the potential usefulness for a therapeutic intervention program. This aim was approached by the following study setting:

1. We assessed emotional analytics upon viewing neutral and alpine stimuli in patients with somatoform, depressive and anxiety disorders and healthy controls. The alpine stimuli depicted individuals while engaged in physical activity in an alpine environment.
2. We investigated whether there was a correlation of emotional analytics with resilience or perceived stress in patients and healthy controls.
3. We measured the amount of self-performed physical activity in an alpine environment as a marker of previous exposure to the depicted stimuli in a natural environment.

## Methods

### Study design

**Fig. 1:**
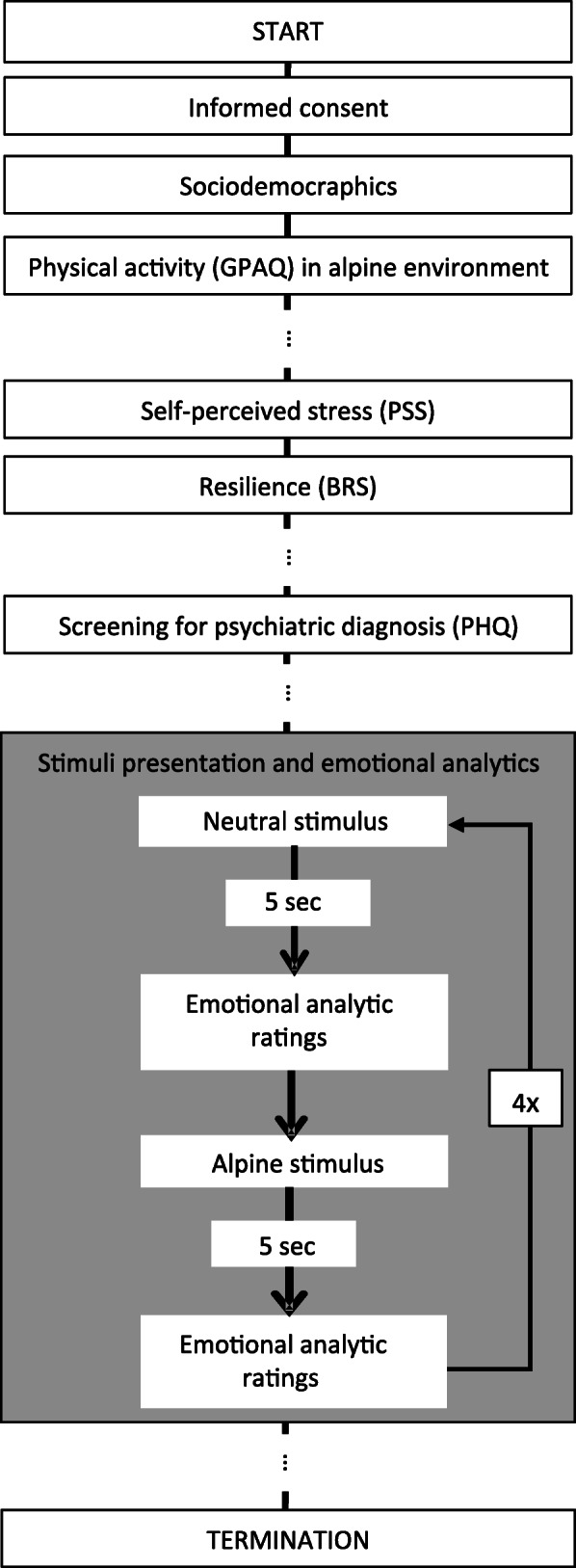
Flow chart of the overall study design including details of the quasi experimental part with presentation of alpine stimuli and emotional analytic ratings (boxed section shaded in grey). Specific questionnaires are indicated. BRS = Brief Resilience Scale, GPAQ = General Physical Activity Questionnaire, PHQ = Patient Health Questionnaire, PSS = Perceived Stress Scale. (…) indicates that there were questionnaires at the indicated point in the study design not analyzed in the current study but in [32]

This is a cross-sectional observational study including a quasi-experimental part (Fig. 1). The whole study was performed online. The first part of the study contained questionnaires, while the second part recorded emotional reaction to visual stimuli. It was not possible to skip one question or a questionnaire. The current data is part of a larger study examining the effect of physical activity in an alpine environment on mental health, part of which has been published [32]. Innsbruck is one of few urban spaces located directly within the Alps and thus allows for easy access to the alpine environment. The ethics commission of the Medical University of Innsbruck reviewed and approved the study protocol. After being informed in detail about the study aims and procedures, participants provided informed consent prior to study participation. Study recruitment was conducted over a four-month period in 2016.

### Participants

**Fig. 2:**
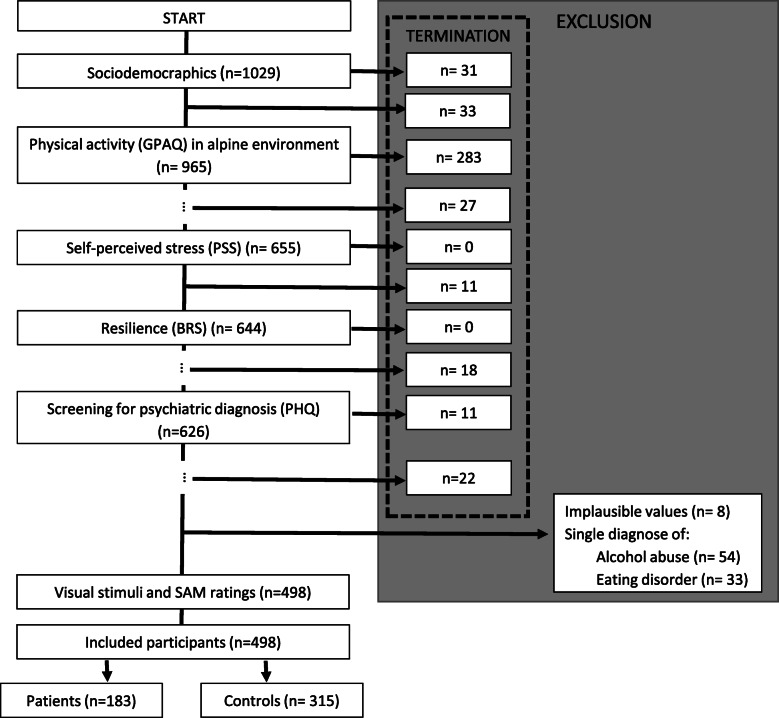
Flowchart of patient and healthy control recruitmen. Excluded cases terminated early, reported implausible values or had a single diagnose of alcohol abuse or eating disorder. Abbreviations: BRS = Brief Resilience Scale, GPAQ = General Physical Activity Questionnaire, PHQ = Patient Health Questionnaire, PSS = Perceived Stress Scale, SAM ratings = Self-Assessment Manikin for emotional analytic ratings. (…) indicates that there were questionnaires at the indicated point in the study design not analyzed in the current study but in [32]

Participants and recruiting procedure are described in [32], participant numbers vary slightly compared to the previous publication due to missing data in individual participants. In brief, a total of 1029 individuals participated in an open online-only survey. They were recruited via email (mailing lists), social media and classified websites or whilst treated at the Department of Psychiatry, Psychotherapy and Psychosomatics (Division of Psychiatry II/Psychosomatic Medicine) at Innsbruck Medical University at the inpatient or outpatient clinic. We included mainly patients with the diagnosis of somatoform, depressive and anxiety disorders. For the present analysis participants who terminated the questionnaire early i.e. prior to the Self-Assessment Manikin (SAM) ratings (missing data *n* = 436, Fig. 2) were excluded from the study. This high drop-out rate was mainly due to the fact that SAM ratings of emotional analytics were performed as the final phase of the questionnaire and it was not possible to skip questions. Comparison of participants terminating early with those included in the data analysis showed that the former were significantly older (mean age ± standard deviation, 33.5 ± 12.1 years vs 29.7 ± 10.1 years, *p* < 0.001, Mann-Whitney U-test) and that a larger proportion of them was female (68.4% vs 61.2%, *p* = 0.017, Chi-square test). Despite statistical significance, the differences in age (effect size d = 0.34) and sex distribution (odds ratio = 1.37) were comparatively small. Furthermore participants that reported implausible values (*n* = 8), screened positively for alcohol abuse only (*n* = 54) or for an eating disorder only (Anorexia nervosa and Bulimia nervosa; *n* = 33) were excluded from the present analysis (Fig. 2). In Anorexia nervosa or Bulimia nervosa it is known that high levels of PA are used as tool for losing weight and therefore are an expression of disease. Therefore, these patients were excluded [3]. There were 4 to 13% missing values for individual SAM ratings. The 498 participants included in the present analysis consisted of two groups. Patients screened positively for mental health disorder on the Patient Health Questionnaire (PHQ, *n* = 183). Participants without positive PHQ screening (*n* = 315) formed the control group (HC).

### Stimuli

**Fig. 3:**
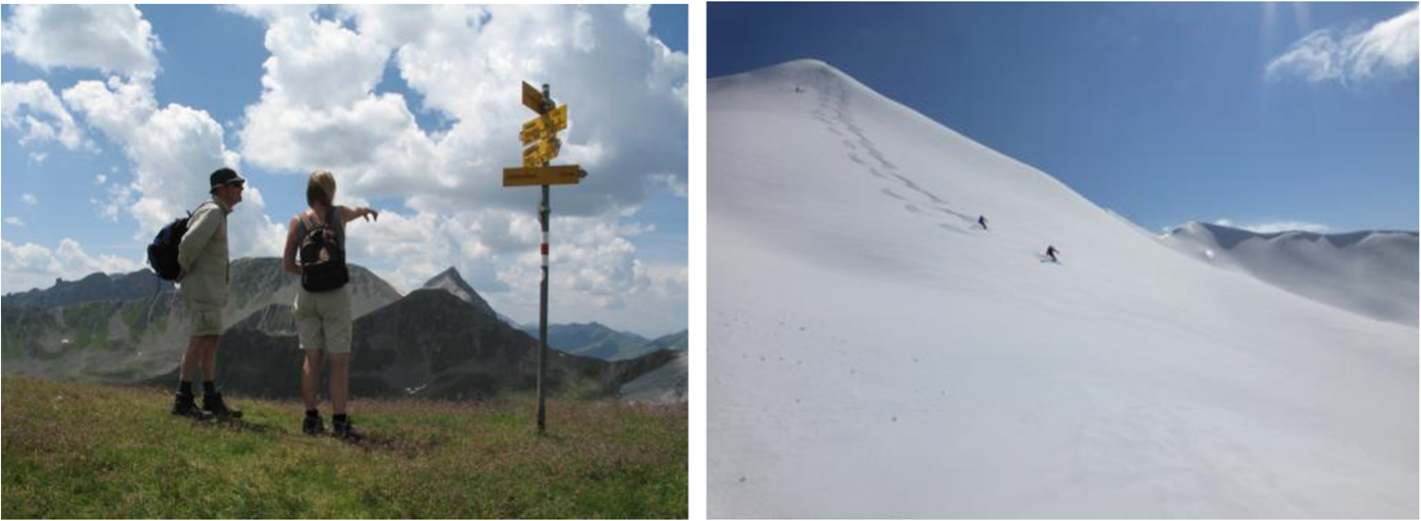
Examples of alpine stimuli depicting individuals performing physical activity in an alpine environment. Neutral stimuli are not depicted since this is not considered good scientific practice for the IAPS picture collection [25]

Stimuli were alternating 5 neutral pictures (re-staged to official International Affective Picture System (IAPS) pictures (slide no. 6150, 7009, 5661, 5500, 7150)) and 5 alpine stimuli (Fig. 3). Neutral pictures displayed figural subjects of daily life (e.g. mug, wall, umbrella). Alpine stimuli displayed alpine environments with individuals performing some sort of physical activity therein (e.g. hiking, biking, skiing). The pictures were presented to all participants in the same order. Two picture stimuli had to be excluded due to considerations related to the displayed content (canyon wall in the neutral stimuli and paraglider in the mountains in the alpine stimuli) and their mean ratings for at least one of the analyzed dimensions ranging two standard deviations outside the mean of the other stimuli in the group. Pictures were displayed for 5 seconds before the page with the emotional analytic ratings appeared. Each stimulus could only be observed once (Fig. 1).

### Measures

Socio-demographic parameters included information on age, sex, education and marital status. Mental health was assessed using the German version of Patient Health Questionnaire [16]. Additionally, open text fields were provided for entering psychiatric diagnoses. Resilience was measured using the Brief Resilience Scale (BRS) [36], self-perceived stress using the Perceived Stress Scale (PSS) [7] and physical activity (PA) using the Global Physical Activity Questionnaire (GPAQ-2) [6]. PA is calculated using metabolic equivalents of tasks (METs) as a unit for energy expense. As proposed by the World Health Organization we classified PA in moderate and vigorous intensity. We adapted the standard questionnaire to measure PA performed in the alpine environment.

To measure emotional response we used the Self-Assessment Manikin (SAM) 9-point Likert-scale. This scale measures emotional analytics in the three dimensions valence, arousal and dominance [25]. The valence scale ranges from a frowning, unhappy (adjectives used in the SAM manual: unhappy, annoyed, unsatisfied, melancholic, despaired, bored; lower values) to a smiling, happy figure (happy, pleased, satisfied, contented, hopeful). The arousal scale displays the lowest value with a calm, eyes-closed figure (relaxed, calm, sluggish, dull, sleepy, unaroused), whilst the highest value is represented by an excited figure (stimulated, excited, frenzied, jittery, wide-awake, aroused). The lowest values in the dominance scale are symbolized by a controlled small figure (controlled, influenced, cared-for, awed, submissive, guided) whilst highest values are represented by a dominant and oversized figure (controlling, influential, in control, important, dominant, autonomous). After presenting a picture for 5 seconds participants were asked to rate their emotional reaction in the three dimensions. For alpine stimuli, we added a fourth dimension asking about ones attraction to the situation, labelled motivational direction. The 9-point Likert-scale ranged from “I don’t want to be in this situation” to “I want to be in the situation”.

### Statistical methods

Metric variables were analyzed for normal distribution prior to applying further statistical tests by assessing their skewness and their kurtosis, considering skewness values > 0.5 or < − 0.5 [27] and kurtosis values > 1 or < − 1 [18] as deviations from a normal distribution requiring non-parametric testing. To compare emotional reactions between overall neutral and alpine pictures we created a mean score for each category. In each category one picture was excluded due to statistical outliers (paraglide in alpine pictures; red wall in neutral pictures). Mean scores were calculated for each emotional dimension per person if at least three scores were completed. Group comparisons (patients vs. HC) were performed using t-test, Mann-Whitney U-test and Chi-square test, depending on the variable type and distribution. As the two groups differed significantly in their age, education, marital status, and work situation, we also performed analyses of covariance with adjustment for these potential confounders. As the emotional analytic ratings displayed missing values (4 to 13%), we performed an additional analysis where missing ratings were replaced by imputed values. The SPSS Missing Value Analysis procedure with Little’s test for missingness completely at random (MCAR) and imputation by expectation-maximization (EM) was used for this purpose [19]. The relationship between resilience, self-perceived stress, PA and emotional analytics was investigated on a descriptive level by means of correlation analysis. Spearman rank correlation coefficients were used as most the variables involved showed deviations from a normal distribution.

## Results

### Sociodemographic characteristics and clinical features

The sociodemographic characteristics of patients and HC are displayed in Table 1. Patients’ diagnoses according to PHQ were in decreasing frequency: somatoform disorder (*n* = 101, 55.2%), major depressive syndrome (*n* = 67, 36.6%), other anxiety syndrome (*n* = 45, 24.6%), panic syndrome (*n* = 36, 19.7%), other depressive syndrome (*n* = 34, 18.6%), alcohol abuse (*n* = 31, 16.9%), binge eating disorder (*n* = 23, 12.6%), bulimia nervosa (*n* = 10, 5.5%) and others (*n* = 2, 1.1%). More than half of the patients (*n* = 100, 51.9%) were diagnosed with more than one mental health disorder, the most prevalent combination was somatoform disorder and major depressive syndrome (*n* = 42, 23.0%).

**Table 1:** Sociodemographic characteristics of patients and healthy controls (adapted with participant numbers for the current analysis from [32])

Variable	Groups		Comparison		
	Patients (*n* = 183)	Controls (*n* = 315)	Test statistics	D.f.	*p*-value
Age in years^a^	36.0 ± 12.8	32.8 ± 11.7	Z = 2.42^c^		0.016
Female gender^b^	117 (63.9)	187 (58.4)	χ^2^ = 1.02^d^	1	0.313
Education^b^	–	–	χ^2^ = 30.989^d^	3	< 0.001
University	41 (22.4)	111 (35.2)		–	–
Secondary school	62 (33.9)	133 (42.2)		–	–
Vocational training	53 (29.0)	34 (10.8)		–	–
Compulsory school and other	27 (14.8)	37 (11.7)		–	–
Marital status^b^	–	–	χ^2^ = 13.699^d^	2	0.001
Single	105 (57.4)	194 (61.6)		–	–
Married	56 (30.6)	110 (34.9)		–	–
Separated/divorced/widowed	22 (12.0)	11 (3.5)		–	–
Employment^b^	–	–	χ^2^ = 66.81^d^	2	< 0.001
Full−/part-time employment	75 (41.0)	177 (56.2)			
In education/study/vocational training	49 (26.8)	122 (38.7)			
Unemployed	59 (32.2)	16 (5.1)			

^a^mean ± standard deviation

^b^absolute number (percent)

^c^test statistic for Mann-Whitney U-test

^d^test statistics for Chi-Square test

### Comparison of resilience, self-perceived stress and emotional analytics in patients and HC

The mean score of the Brief Resilience Scale (BRS) was significantly lower in patients than in HC (Mann-Whitney U-test, *p* < 0.001; Table 2). Furthermore the total score of the Preceived Stress Scale (PSS) was significantly higher in patients than in HC (Mann-Whitney U-test, *p* < 0.001; Table 2).

**Table 2:** Resilience, self-perceived stress and emotional analytics (SAM ratings) in patients and controls

Variable	Group		Comparison		
	Patients (*N* = 183) Mean ± SD	Controls (*N* = 315) Mean ± SD	Test statistics	Effect size, d	*p*-value^a^
**Resilience (BRS score)**	2.78 ± 0.85 ↓	3.76 ± 0.66	Z = -11.84	−1.33	< 0.001
**Stress (PSS score)**	9.53 ± 3.61 ↑	4.73 ± 2.50	Z = -13.47	1.62	< 0.001
**SAM Rating**					
Neutral pictures					
Valence	5.09 ± 1.06 ↓	5.65 ± 1.21	Z = -4.696	− 0.48	< 0.001
Arousal	4.13 ± 1.31 ↑	3.38 ± 1.23	Z = 5.848	0.60	< 0.001
Dominance	4.78 ± 1.08 ↓	5.13 ± 1.35	Z = -2.312	− 0.15	0.021
Alpine pictures					
Valence	6.99 ± 1.68 ↓	7.85 ± 1.12	Z = -5.661	−0.64	< 0.001
Arousal	5.01 ± 1.76	5.17 ± 1.94	Z = -1.218	− 0.09	0.223
Dominance	5.85 ± 1.52 ↓	6.42 ± 1.58	Z = -3.655	−0.37	< 0.001
Attraction	6.62 ± 2.10 ↓	7.52 ± 1.48	Z = -4.106	−0.52	< 0.001
Comparison (Alpine>Neutral)					
Valence	1.91 ± 1.80 ***	2.19 ± 1.42 ***	Z = -1.466	−0.18	0.143
Arousal	0.87 ± 2.11 ↓**	1.79 ± 1.91 ***	Z = -4.741	−0.46	< 0.001
Dominance	1.09 ± 1.61 ***	1.29 ± 1.67 ***	Z = -1.465	−0.12	0.143

*Abbreviations*: *BRS* Brief Resilience Scale 13, *PSS* Perceived Stress Scale, *SAM *Self-Assesment Manikin, *SD* standard deviation

^a^*p*-values were calculated using Mann-Whitney U-test

↑ Significantly higher scores in patients than in healthy controls.

↓ Significantly lower scores in patients than in healthy controls.

** Difference “alpine – neutral” significantly greater than 0, Z = 3.25, *p* < 0.01

*** Difference “alpine – neutral” significantly greater than 0, always Z ≥ 4.5, *p* < 0.001

Comparing the mean emotional analytics score in neutral and alpine stimuli, patients reported significantly lower values for valence (both ps < 0.001) indicating that they felt less happy than HC, and dominance (neutral: *p* = 0.021, alpine: *p* < 0.001; Table 2) indicating that they felt less in control than HC. Arousal when viewing neutral stimuli was significantly higher (*p* < 0.001) for patients indicating that they felt more aroused or jittery than the HC at baseline. In alpine pictures the difference in arousal was not significant between patients and HC (*p* = 0.223; Table 2). In the fourth dimension asking about attraction to the displayed alpine situation, the mean score was significantly lower in patients as in HC (*p* < 0.001 Table 2) although both groups showed a high attraction to the alpine stimuli. All statistically significant differences in Table 2 remained significant when adjusting for age, education, marital status, and work situation by analysis of covariance. Missing value analysis for emotional analytics revealed that SAM ratings were not missing completely at random (Little’s test, χ^2^ = 3607.5, d.f. = 3314, *p* < 0.001). Replacement of missing emotional analytics ratings by the EM imputation method led to comparable results as the analysis without replacement. Mean ratings changed by less than 0.1 in both groups. Moreover, all significant group differences were retained.

To measure the effect of the alpine stimuli normalized to the neutral baseline, we evaluated the difference of each emotional dimension between alpine and neutral pictures. The comparison alpine > neutral stimuli was significantly greater than 0 for both patients and HC indicating a positive effect of alpine stimuli on emotional analytics. For valence and dominance this comparison of alpine > neutral stimuli did not differ significantly between patients and HC (Table 2). For arousal the difference was significantly smaller in patients than in HC due to higher baseline arousal values in patients (*p* < 0.001; Table 2).

### Correlation between resilience, self-perceived stress, physical activity in an alpine environment and emotional analytics

For the correlation analysis between resilience, self-perceived stress and emotional response, we combined the patient and HC group to one total sample. Resilience correlated positively in both neutral and alpine stimuli with the emotional analytics for valence, dominance and attraction (all ps < 0.001, Table 3) indicating that greater resilience was associated with higher emotional ratings. Self-perceived stress correlated negatively with valence, dominance and attraction in both neutral and alpine stimuli (all ps < 0.05; Table 3) demonstrating that higher stress levels were associated with lower emotional ratings (Table 3).

**Table 3:** Correlation of emotional analytics (SAM) with resilience, self-perceived stress and PA in alpine environment

		Total sample (*n* = 498)		
		BRS	PSS	PA in alpine environment (MET)
Neutral pictures				
Valence	r_s_	0.188***	−0.249***	0.081
	p	0.000	0.000	0.078
Arousal	r_s_	−0.183***	0.187***	−0.091
	p	0.000	0.000	0.051
Dominance	r_s_	0.227***	−0.150**	− 0.021
	p	0.000	0.002	0.656
Alpine pictures				
Valence	r_s_	0.303***	−0.276***	0.440***
	p	0.000	0.000	0.000
Arousal	r_s_	0.073	−0.096*	0.225***
	p	0.121	0.040	0.000
Dominance	r_s_	0.209***	−0.172***	0.277***
	p	0.000	0.000	0.000
Attraction	r_s_	0.222***	−0.172***	0.413***
	p	0.000	0.000	0.000
Comparison (Alpine>Neutral)				
Valence	r_s_	0.125**	−0.043	0.316***
	p	0.007	0.358	0.000
Arousal	r_s_	0.175***	−0.188***	0.266***
	p	0.000	0.000	0.000
Dominance	r_s_	0.043	−0.025	0.278***
	p	0.368	0.604	0.000

*Abbreviations*: *MET* metabolic equivalents, *BRS* Brief Resilience Scale, *PA* physical activity, *PSS* Perceived Stress Scale

r_s_: Spearman rank correlation coefficient, p: *p*-value, *p < 0.05, **p < 0.01. ***p < 0.001

Arousal while viewing neutral pictures correlated in an inverse way: negatively with resilience and positively with perceived stress. Subanalyses demonstrated that this was mostly due to patients´ values (not shown). This demonstrates that individuals with low resilience and high levels of stress feel more aroused or jittery at baseline compared to resilient individuals who feel calmer when viewing neutral stimuli. Physical activity in an alpine environment correlated positively with all four emotional analytics in alpine stimuli (all *p* < 0.001), whilst there was no significant correlation with neutral stimuli (Table 3).

## Discussion

In the present study we evaluated the effect of viewing alpine stimuli on emotional analytics in patients with somatoform, depressive and anxiety disorders and healthy controls. The major findings were: 1) the emotional analytics valence and dominance were significantly lower in patients compared to HC for both alpine and neutral stimuli. Baseline arousal when viewing neutral stimuli was significantly higher in patients, 2) the emotional analytic scores were significantly higher for alpine compared to neutral pictures for patients as well as for HC, 3) Emotional analytics of alpine pictures correlated positively with resilience and physical activity in an alpine environment and inversely with perceived stress.

### Resilience and perceived stress in patients with psychosomatic disorders

In patients with somatoform, depressive and anxiety disorders we observed lower levels of resilience and higher levels of perceived stress compared to HC. These findings are in line with previous studies showing that patients with mental disorders often lack strategies of a resilient mindset, which can improved during recovery [26]. Likewise perceived stress has been shown to be elevated in states of emotional-ill being [22]. Impaired resilience and higher perceived stress, are part of the current vulnerability-stress-model of psychosomatic disorders [12].

### Emotional analytics in response to neutral and alpine stimuli in patients with somatoform, depressive and anxiety disorders

We found lower levels of valence and dominance in patients than in HC over all (neutral and alpine) stimuli. The lower levels of valence (i.e. more unhappy) reflect the fact that our largest subgroup in our patient group was „depressive disorders” (55.2%). This confirms previous studies showing that patients suffering from depression tend to show lower levels of valence as they describe a feeling of numbness und joylessness in their lives [9]. A dysfunction in emotional processing might be the underlying pathophysiological concept [23]. Viewing alpine stimuli lead to a comparable increase in valence (feeling happier) and dominance (feeling more in control) in patients and controls. Baseline arousal was higher in the patients than HC a finding previously described in individuals with depressive symptoms [15]. This led to a significantly smaller increase in arousal between neutral and alpine stimuli for patients than controls.

### Association of resilience, perceived stress and emotional analytics

The association of resilience and perceived stress with emotional analytics was found not only in patients with somatoform, depressive and anxiety disorders but also in healthy controls. This underlines the theory that there is a continuum of health and disease also for somatoform, depressive and anxiety disorders, and that mechanisms of overtly ill patients are also present in individuals with sub-syndromal forms of psychosomatic disorders pointing towards general mechanisms of mental health [24]. The inverse correlation of arousal while viewing neutral pictures (negatively with resilience and positively with perceived stress) were mostly due to patients´ values: They are more jittery or aroused at baseline which fits well with their predominant diagnoses of somatoform, depressive and anxiety disorders [21].

### The effect of alpine stimuli on emotional analytics

The effect the alpine environment on mental health has rarely been researched to date, most studies where performed on other natural environments. In the present study we found that both patients and HC reacted to alpine stimuli in form of a significant increase in valence, arousal and dominance compared to neutral stimuli. This finding of a positive impact on emotional analytics is in line with previous studies evaluating psychological and physical reactions to visual natural stimuli. Comparing reactions to urban with those to natural scenery a significant increased positive affect in emotional response could be found in nature condition only using virtual reality stimuli [42]. The restorative effect of the natural environment, even if only present within visual stimuli, might be explained by a reduction in stress levels induced by exposure to views of nature [42]. Patients and HC showed higher emotional analytics for valence and dominance, but we also detected an increase in arousal in response to the alpine stimuli. This is in contrast with several studies pointing towards relaxation and tranquility felt while viewing natural environment [10]. One possible explanation of our diverging finding is that most of the alpine pictures shown in this study displayed physically active persons (e.g. downhill skiing). Comparable data were published by IAPS showing high arousal ratings in the SAM scale when viewing stimuli of physically active persons in alpine surroundings [25]. People living in perceived safe, lively and beautiful neighborhoods were more likely to engage in PA, and people living in perceived boring and depressing neighborhoods were less likely to engage in PA [44]. Multilevel modeling results showed that after controlling for depressive symptoms at baseline, symptoms decreased in neighborhoods where physical environment and social environment were better [45].

### The effect of physical activity in an alpine environment on mental health

Physical activity by itself and especially when performed in an outdoor/green/alpine environment is known to improve mental health. Few pilot studies could confirm the positive effect of the alpine environment when performing physical activity [29, 32, 37]. This is in line with our finding that self-performed physical activity (METs) correlates with higher valence and dominance felt by participants after viewing alpine but not neutral stimuli. Conversely, some studies did not detect any differences in affective response when comparing alpine to indoor physical exercise [30]. Furthermore, no effect of anthropogenic elements in the alpine environment on acute stress-related physiological responses was found [31]. Though importantly the latter studies as well as the present one showed a positive correlation of outdoor physical activity on parameters of mental well-being.

### Limitations

The main limitation of the study is that in a survey study no causal relationship between the emotional analytics and mental health can be obtained. Furthermore, the exposure in our study was applied in form of visual stimuli instead of actually spending time in an alpine environment. The present study does not allow the differentiation which components of viewing alpine environment lead to the observed positive effects on the emotional analytics. This was a cross sectional study which cannot give any evidence about the long term effects on emotional analytics. Due to the spread of the study invitation via social media, flyers, classified websites and mailing list, we cannot report the response rate.

### Conclusion and consequences for clinical practice

Therapeutic programs for patients with somatoform, depressive and anxiety disorders should contain physical activity and according to our results, also consider taking the effect of nature into account. The results from the current study indicate that patients with somatoform, depressive and anxiety disorders have a positive attitude towards physical activity in an alpine environment and that emotional analytics such as valence and dominance increase in patients and HC in a comparable manner. Practical strategies to implement such programs should be discussed. Obvious barriers to the implementation of such programs are primarily of a financial origin, since in our medical system money for medications and inpatient hospital stays is readily available while therapeutic programs including physical activity in an alpine environment are not financed by public healthcare. To further elucidate the effect of physical activity in an alpine environment on mental health longitudinal intervention studies are needed. The current study indicates that such studies could be promising.

## Data Availability

Data are available from the first author upon request.
